# Effectiveness of a Novel Nutraceutical Compound Containing Red Yeast Rice, Polymethoxyflavones and Antioxidants in the Modulation of Cholesterol Levels in Subjects With Hypercholesterolemia and Low-Moderate Cardiovascular Risk: The NIRVANA Study

**DOI:** 10.3389/fphys.2019.00217

**Published:** 2019-03-11

**Authors:** Paolo Cimaglia, Francesco Vieceli Dalla Sega, Francesco Vitali, Veronica Lodolini, Davide Bernucci, Giulia Passarini, Francesca Fortini, Luisa Marracino, Giorgio Aquila, Paola Rizzo, Roberto Ferrari, Gianluca Campo

**Affiliations:** ^1^Cardiovascular Institute, Azienda Ospedaliero-Universitaria di Ferrara, Ferrara, Italy; ^2^Maria Cecilia Hospital, GVM Care & Research, Cotignola, Italy; ^3^Department of Medical Sciences, University of Ferrara, Ferrara, Italy

**Keywords:** red yeast rice, nutraceutical compound, hypercholesterolemia, cardiovascular prevention, endothelial function

## Abstract

**Background:** Red yeast rice supplements are broadly accepted as treatment for dyslipidaemia in subjects without high cardiovascular (CV) risk. Their effect on lipid profile is well known, but few data are available on their effect on endothelial function.

**Objectives:** To study the effect of a novel nutraceutical compound (NC) containing low monacolin K dose, polymethoxyflavones and antioxidants on lipid profile, endothelial function and oxidative stress.

**Methods:** Fifty-two subjects with low-moderate CV risk and dyslipidaemia (according to European guidelines) were enrolled and treated for 8 weeks with the NC. Blood samples were collected at baseline and at the end of treatment to assess changes in lipid profile, endothelial function and oxidative stress. The primary endpoint was the reduction of low density lipoprotein (LDL) cholesterol. Endothelial function was assessed through measurement of rate of apoptosis and nitric oxide (NO) production in human umbilical vein endothelial cells (HUVECs) treated with subject’s serum. High-sensitivity C-reactive protein, 4-hydroxynonenal (HNE) and oxidized LDL (oxLDL) were markers of oxidative stress.

**Results:** Fifty subjects completed the study. The treatment caused a significant decrease in LDL (−15.6%, *p* < 0.001), oxLDL (−21.5%, *p* < 0.001), total cholesterol (TC), triglycerides, and ApoB. Apoptosis rate of HUVECs significantly decreased (−15.9%, *p* < 0.001). No changes were noted for NO levels and 4-HNE protein adducts. The reduction of the apoptosis rate was correlated to the reduction of oxLDL.

**Conclusion:** An 8-week treatment based on a novel NC containing low manocolin K dose, polymethoxyflavones and antioxidants improved lipid profile in subjects with dyslipidaemia and low-moderate CV risk. Secondarily, we observed an improvement in surrogate markers of endothelial function that may result from the reduction of oxLDL (Registered at www.clinicaltrials.gov, NCT03216811).

## Introduction

Dyslipidaemia represents an important CV risk factor. Reduction of low-density lipoprotein cholesterol (LDL) leads to a reduction of major adverse cardiovascular events (MACE), independently of the drug used (Silverman et al., 2016).

In primary prevention, according to international guidelines, a healthy lifestyle represents the first approach to dyslipidaemia, in case of unsuccess, drugs are needed. When drugs are needed, statins are the lipid-lowering treatment of choice due to the large evidence of efficacy and safety in reducing both LDL and MACE (Cholesterol Treatment Trialists’ (CTT) Collaboration et al., 2010; Cholesterol Treatment Trialists’ (CTT) Collaborators et al., 2012). However, statins are cardioprotective even in the presence of normal LDL cholesterol levels due to their pleiotropic effect (Sever et al., 2003; Oesterle et al., 2017). In the last few years, red yeast rice NC (containing monacolin K) have been accepted as possible alternatives to statins (Pirro et al., 2016; Johnston et al., 2017; Poli et al., 2018; Zhang et al., 2018). Current European guidelines state that NC may be considered in individuals with elevated plasma cholesterol concentrations who do not qualify for drug treatment with statins in view of their global CV risk (Catapano et al., 2016). NC are also an alternative for subjects with intolerance to or not willing to take statins (Banach et al., 2018). Several NC are available on the market, and the common denominator is monacolin K, whose activity on 3-hydroxy-3-methylglutaryl coenzyme A (HMG-CoA) reductase leads to the reduction of endogenous synthesis of cholesterol. Beside monacolin K, NC contain other molecules (e.g., berberin, policosanols, plant sterols, vitamins, and antioxidants) in various concentrations and compositions. These cofactors have lipid-lowering activities that are in synergy with monacolin K action, and the effectiveness of the compounds is generally assessed in term of LDL reduction. Few studies investigated the effect of some compounds on endothelial function, through *in vivo* testing (Affuso et al., 2010; Trimarco et al., 2015; Cicero et al., 2016a,b, 2017; Esposito et al., 2018), analysis of endothelial damage biomarkers (Cicero et al., 2013; Derosa et al., 2014; Hermans et al., 2017) or *in vitro* experiments (Lin et al., 2011).The NC to optimize cholesterol, endothelial and inflammatory parameters in subjects with hypercholesterolemia and low to moderate CV risk (NIRVANA) study was designed to test the effect of a novel NC in individuals with dyslipidaemia and low to moderate CV risk, in terms of reduction of cholesterol levels, but also in terms of improvement of endothelial function, and oxidative stress. The main novelty of the tested composition, compared to others, is the presence of polymethoxyflavones from tangerine extract, phenolic acids and flavonoids from Ipomoea batatas (sweet potato) extract, and hydroxytyrosol from olive fruit extract.

Polymethoxyflavones (citrus flavonoids) have anti-inflammatory and anti-atherosclerosis activities (Li et al., 2009), while phenolic compounds from Ipomoea batatas, and hydroxytyrosol have antioxidant activity (Wang et al., 2016; Tejada et al., 2017). It is conceivable that the single components of the combination, by acting with different mechanism of action, could have a beneficial role on endothelial function.

Another important aspect of the tested NC is the dosage of monacolin K (3 mg), which was significantly lower than the 10 mg dose recommended in 2011 by the (European Food Safety Authority (EFSA), 2011). Such a low dose of monacolin K has been proven effective in the reduction of LDL (Heinz et al., 2016), and it is potentially safer than the 10 mg dose. This is a relevant aspect given the recent scientific opinion by EFSA on the safety of monacolins in red yeast rice (Younes et al., 2018).

## Materials and Methods

The NIRVANA is an investigator-initiated, prospective, single-center, interventional study. The Ethical Committee of Ferrara approved the study protocol in June 2017. All subjects gave written informed consent to participate. The study is registered at clinicaltrials.gov with the identifier NCT03216811.

### Inclusion and Exclusion Criteria

Inclusion criteria were: (i) age > 18 years; (ii) ability to provide informed written consent; (iii) low-moderate CV risk according to SCORE (Piepoli et al., 2016), plus one of the following criteria (based on European guidelines on dyslipidaemia): LDL > 190 mg/dl if SCORE < 1% or LDL > 100 mg/dl if SCORE 1–4% (Catapano et al., 2016). Exclusion criteria were: high CV risk (SCORE ≥ 5%), known CV disease, diabetes, creatinine clearance < 60 ml/min, familial hypercholesterolemia, treatment with any lipid-lowering product in the previous 4 weeks, any concomitant chronic disease, intolerance to NC, high alcohol consumption, pregnancy, and breastfeeding.

### Study Design

Subjects were recruited between June and October 2017 in the Prevention Center of the University of Ferrara (Ferrari, 2017; Ferrari and Guardigli, 2017, 2018; Ferrari and Cimaglia, 2018a,b; Ferrari et al., 2018). During the screening visit, study physicians assessed the patients’ eligibility and widely discussed CV primary prevention and the importance of lifestyle changes concerning smoking, physical activity and a healthy diet. After the screening visit, a 4-week run-in period was started. During this period, subjects were strongly encouraged to follow the suggestions and the recommendations discussed in the screening visit. At the inclusion visit, eligibility was reassessed. If inclusion and exclusion criteria were satisfied, the subject was included and the NC was administered. After 8 weeks, the end of study visit was performed. During screening, inclusion and end of treatment visits the following parameters were assessed: blood pressure, heart rate, body weight, waist and hip circumferences.

### Nutraceutical Compound

The NC tested was Cardiovis^®^Colesterolo (Bios Line, Padova, Italy). Each capsule consisted of red yeast rice containing 3 mg of monacolin K, polymethoxyflavones from tangerine extract (mainly nobiletin and tangeretin), hydroxytyrosol from olive fruit extract, phenolic acid, and flavonoids from Ipomoea batatas extract, vitamin E and coenzyme (Table 1).

**Table 1:** Chemical composition of a capsule of nutraceutical compound.

Red rice fermented with *Monascus purpureus* titrated to 3% in monacolin K	3 mg
Tangerine extract titrated to 60% in polymethoxyflavones	24 mg
Olive fruit extract titrated to 12% in hydroxytyrosol	3 mg
Ipomoea batatas extract	160 mg
Vitamin E	10 mg
Coenzyme Q10	5 mg

Before production, titration of monacolin K in the red rice was checked by an external company. Furthermore, the quality of each lot was determined in terms of bacterial, yeasts and molds contamination.

All subjects included in the study received a single daily dose, that was prescribed to be taken in the evening. Treatment adherence was assessed by counting the number of pills returned at the end of study visit.

### Blood Samples

Venous blood samples were collected at baseline (before starting treatment) and at the 8-week visit from an antecubital vein using a 21-gauge needle. All subjects underwent blood sampling after a 12-h fasting. TC, high-density lipoprotein (HDL), triglycerides, apolipoprotein A1 (ApoA1), apolipoprotein B100 (ApoB), creatinine, liver aminotransferases, creatinine phosphokinase and high-sensitivity c-reactive protein (hs-CRP) were measured by standard enzymatic techniques. The LDL levels were calculated according to the Friedewald formula (Friedewald et al., 1972).

### Endothelial Function

The endothelial function was evaluated measuring two surrogate markers of endothelial function: rate of apoptosis and NO production in HUVECs treated with serum from subjects as previously described (Campo et al., 2017; Pavasini et al., 2017; Vieceli Dalla Sega et al., 2018). Briefly, HUVECs were incubated for 48 h with growth media containing 20% serum from subjects. The rate of apoptosis was determined by flow cytometry after staining the cells with annexin V and propidium iodide. It was expressed as percentage (%) of annexin V positive cells compared to the total number of cells. Each experiment was performed in triplicate and rate of apoptosis was the mean of the three experiments. The levels of NO were measured with the fluorogenic NO probe DAF-FM-DA. Fluorescence was quantified by flow cytometry and the NO levels were expressed as mean fluorescence.

### Oxidative Stress

The oxidative stress was assessed by measuring oxLDL and 4- HNE protein adduct in serum. OxLDL was measured by ELISA (oxLDL ELISA oxidized human, Cell Biolabs) according to the manufacturer’s indication. The amount of oxLDL was expressed as arbitrary unit per ml serum. 4-HNE protein adduct was quantified by ELISA (Oxiselect^TM^ HNE, Cell Biolab-competitive ELISA kit) according to the manufacturer’s instructions. 50 μL of serum was added to each well of the HNE conjugate coated plate, and the amount of 4-HNE protein adduct was expressed as μg/ml equivalent to the 4-HNE bovine serum albumin (BSA) standard.

### Outcome

The primary outcome was a change in the LDL cholesterol values after 8 weeks of treatment with the NC. Secondary outcomes included 8-week change of: TC, HDL, triglycerides, non-HDL cholesterol, Apo A1, ApoB, hs-CRP, oxLDL, 4-HNE, rate of HUVECs apoptosis, and NO levels. All subjects who were enrolled in the study and receiving at least one dose of the compound were included in the safety analyses. Any potential side effect was recorded.

### Statistical Analysis

Based on previous studies, we assumed that the treatment would decrease LDL by 10%. Accordingly, at least 40 subjects were required for 80% power and a two-sided-value of 0.05. Continuous data were tested for normal distribution with the Kolmogorov–Smirnov test. The variables normally distributed were presented as mean ± SD, otherwise as median and interquartile range (IQR). For the comparison of parameters before and after treatment, the differences were tested for normality. Paired *t*-test or Wilcoxon test were used as appropriate. Of note, the LDL values (primary outcome) were normally distributed. Categorical variables were summarized in terms of numbers and percentages and were compared by using the two-sided Fisher’s exact test. To better describe the response to NC, we prespecified subgroups (sex: male vs. female; metabolic syndrome as defined by Alberti et al. (2009): yes vs. no; c-reactive protein:< vs. ≥2 mg/L; baseline LDL:< vs. ≥ the median value; waist-hip ratio (WHR):< vs. ≥ World Health Organization cut-offs) where the LDL change was analyzed. A *p*-value was considered significant if <0.05. All analyses were performed with STATISTICA 8 (Statsoft Inc, Tulsa, Okla, United States).

## Results

**FIGURE 1:**
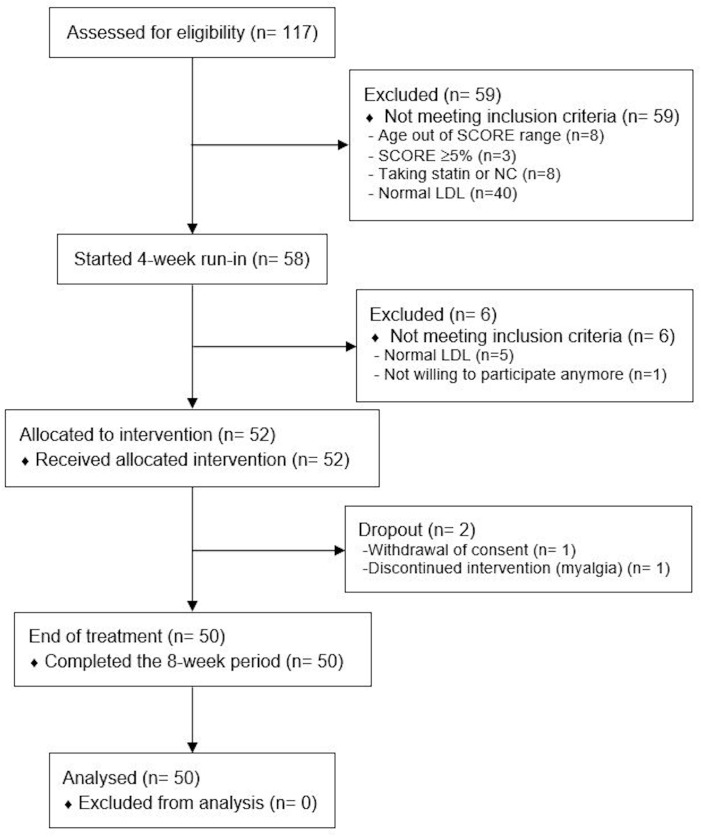
Study flow-chart. SCORE, systemic coronary risk estimation; NC, nutraceutical compound; LDL, low density lipoprotein.

From June to October 2017, 117 subjects were screened (Table 2). Fifty-nine (50%) resulted not eligible or declined to participate. Fifty-eight subjects entered the 4-week run-in period. At the end of the run-in period, 52 subjects satisfied all inclusion and exclusion criteria and were enrolled. NC was regularly taken by 50 subjects (Figure 1 and Table 2). Table 2 shows the main characteristics of study population. Mean age was 56 ± 5 years and 44% were men. Mean LDL was 164 ± 26 and 83% of subjects were at moderate risk according to the SCORE (1–4% risk at 10 years). There were no significant differences of all clinical and laboratory parameters before and after the run-in period. As shown in Figure 1, only two (4%) individuals did not complete the 8-week treatment period. One person stopped treatment for myalgia and one withdrew the consent for personal reasons. Weight, waist circumference and blood pressure did not change during the treatment period (data not shown).

**Table 2:** Characteristics of the study population.

	Screened population (*n* = 117)	Study population (*n* = 52)
**Clinical characteristics**		
Age (years)	54.1 ± 9.6	55.7 ± 4.8
Male sex, no. (%)	73 (62.4)	23 (44.2)
BMI (Kg/m2)	26.3 ± 3.8	25.8 ± 4.0
Hypertension, no. (%)	21 (17.9)	7 (13.5)
Family history of CVD, no. (%)	13 (11.1)	7 (13.5)
Current smoker, no. (%)	16 (13.7)	9 (17.3)
Previous smoker, no. (%)	41 (35.0)	16 (30.8)
Physical inactivity, no. (%)	31 (26.5)	14 (26.9)
Systolic BP (mmHg)	122 (115–130)	125 (120–130)
Diastolic BP (mmHg)	75 (68–80)	80 (70–80)
Heart rate (bpm)	66.1 ± 10.2	64.5 ± 9.7
Waist circumference (cm)	93.8 ± 12.2	92.2 ± 11.9
WHR	0.90 (0.87–0.96)	0.86 (0.83–0.92)
Metabolic syndrome, no. (%)	37 (31.6)	16 (30.8)
**Laboratory data**		
Total cholesterol (mg/dl)	207.2 ± 32.2	246.2 ± 27.1
HDL (mg/dl)	57.0 ± 13.3	59.3 ± 13.6
Triglycerides (mg/dl)	95.0 (70.0–131.0)	101.0 (75.5–163.5)
LDL (mg/dl)	128.9 ± 27.5	164.0 ± 26.1
Creatinine clearance (ml/min)	90.0 (79.7–102.7)	90.9 (75.6–100.4)
Creatinine (mg/dl)	0.89 ± 0.13	0.84 ± 0.19
**10-year cardiovascular risk**		
SCORE		
<1%, no. (%)	26 (22.2)	9 (17.3)
1–5%, no. (%)	80 (68.4)	43 (82.7)
Relative risk		
1 no. (%)	68 (58.1)	14 (26.9)
2 no. (%)	38 (32.5)	23 (44.2)
>2 no. (%)	10 (8.5)	15 (28.9)
**Cardiovascular therapy**		
Aspirin, no. (%)	3 (2.6)	4 (7.7)
ACEi/ARB, no. (%)	19 (16.2)	6 (11.5)
Beta-blocker, no. (%)	1 (0.9)	1 (1.9)
Other antihypertensive drugs, no. (%)	9 (7.7)	3 (5.8)

*The variables normally distributed are presented as mean ± SD, otherwise as median (IQR). BMI, body mass index; CVD, cardiovascular disease; BP, blood pressure; WHR, waist-hip ratio; HDL, high density lipoprotein; LDL, low density lipoprotein; SCORE, systemic coronary risk estimation; ACEi, angiotensin converting enzyme inhibitor; ARB, angiotensin II receptor blocker.*

### Lipid Profile

**FIGURE 2:**
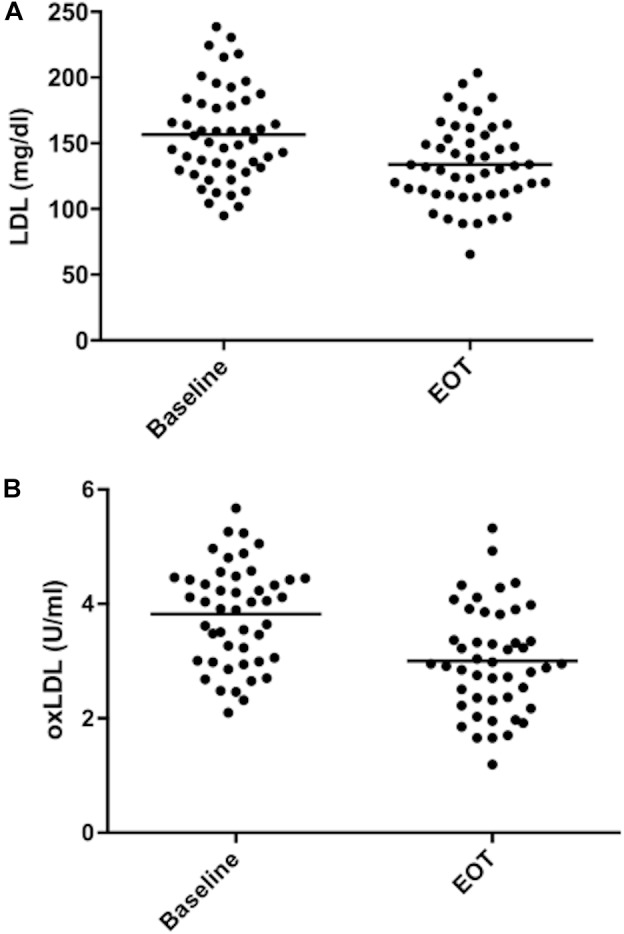
Low density lipoprotein and oxidized low density lipoprotein values during the study. (**A**) LDL values. (**B**) oxidized LDL values. Mean values are shown as bars. EOT, end of treatment.

Table 3 shows the effect of treatment on the lipid profile. After 8 weeks there was a significant decrease of LDL (−15.6%, *p* < 0.001) (Figure 2A), concomitant with a significant decrease of TC, non-HDL cholesterol, triglycerides, ApoB, and five different lipoprotein ratios (Table 3). There was also a significant increase in ApoA1 (+2.1%, *p* = 0.01) and a trend for HDL raising (+3.2%, *p* = 0.057) (Table 3).

**Table 3:** Lipid profile and safety parameters before and after the treatment.

	Baseline (*n* = 52)	EOT (*n* = 50)	*Treatment difference*		*P*
LDL (mg/dl)	156.6 ± 35.4	133.9 ± 30.3	−24.5 ± 22.8	−15.6%	<0.001
TC (mg/dl)	241.0 ± 40.4	216.4 ± 36.2	−24.7 ± 23.9	−10.2%	<0.001
HDL (mg/dl)	56.0 ± 15.3	57.2 ± 14.1	1.8 ± 6.7	3.2%	0.057
Triglycerides (mg/dl)	107.5 (82.3–171.3)	100.5 (78.0–148.3)	−13.5 (−40.5 – 18.25)	−12.6%	0.032
nonHDL cholesterol (mg/dl)	185.0 ± 39.0	159.1 ± 35.2	−26.5 ± 24.3	−14.3%	<0.001
TC/HDL	4.6 ± 1.3	4.0 ± 1.0	−0.68 ± 0.72	−14.8%	<0.001
nonHDL/HDL	3.58 ± 1.32	2.96 ± 0.99	−0.68 ± 0.72	−19.0%	<0.001
LDL/HDL	2.9 ± 0.9	2.5 ± 0.7	−0.5 ± 0.5	−17.2%	<0.001
AIP	0.01 ± 0.36	−0.06 ± 0.28	−0.07 ± 0.21	−7%	0.025
ApoB (mg/dl)	112.2 ± 21.1	94.3 ± 19.5	−18.5 ± 13.1	−16.5%	<0.001
ApoA1 (mg/dl)	142.7 ± 17.9	144.6 ± 17.4	3.0 ± 7.9	2.1%	0.01
ApoB/ApoA1	0.80 ± 0.19	0.66 ± 0.17	−0.15 ± 0.09	−18.8%	<0.001
AST (U/L)	22.4 ± 4.9	23.3 ± 6.2	0.9 ± 3.9	4%	0.103
ALT (U/L)	19.6 ± 7.3	20.6 ± 7.7	1.0 ± 4.5	5.1%	0.120
CK (U/L)	144.5 ± 84.5	139.2 ± 71.3	−5.4 ± 50.7	−3.7%	0.458
Creatinine (mg/dl)	0.82 ± 0.13	0.83 ± 0.14	0.01 ± 0.06	1.2%	0.181

*The variables normally distributed are presented as mean ± SD, otherwise as median (IQR). EOT, end of treatment; AIP, atherogenic index of plasma [log(triglycerides/HDL)].*

### Prespecified Subgroups Analysis

**FIGURE 3:**
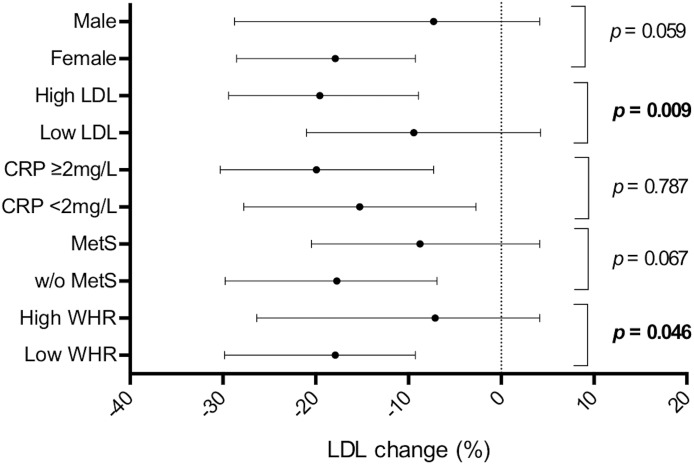
Low density lipoprotein reduction in different subgroups. LDL reduction is shown as median and interquartile range. *P*-values refer to the difference in the subgroups shown on the left. CRP, c-reactive protein; MetS, metabolic syndrome; WHR, waist-hip ratio.

Figure 3 shows the differences in LDL reduction among the subgroups. The median value of baseline LDL was 152.8 mg/dl. The response to NC was stronger in the subgroup with higher baseline LDL compared to subjects with lower values (−19.7% vs. –9.1%, *p* = 0.009). Furthermore, subjects with normal values of WHR had a greater LDL reduction compared to subjects with abnormal values (−18.2% vs. −10.0%; *p* = 0.046). Even though there was a trend for a better response to NC in female and in subjects without metabolic syndrome, the differences in the other couples of subgroups were not statistically significant. The effect of the NC on triglycerides, HDL, ApoB and ApoA1 in different subgroups is shown in Supplementary Material.

### Endothelial Function

The eight-week treatment with NC significantly reduced the rate of apoptosis in HUVECs (−15.9%, *p* < 0.001) (Table 4) but did not affect the NO levels (−1.2%, *p* = 0.6).

**Table 4:** Parameters regarding endothelial function and oxidative stress.

	Baseline (*n* = 52)	EOT (*n* = 50)	*Treatment difference*		*P*
Apoptosis rate (%)	6.9 ± 1.6	5.8 ± 1.3	−1.1 ± 1.0	−15.9%	<0.001
Nitric oxide (FAU)	25.7 ± 4.6	25.4 ± 4.6	−0.3 ± 4.1	−1.2%	0.626
4-HNE (U/ml)	70.9 ± 51.8	78.6 ± 54.2	7.7 ± 30.3	10.9%	0.089
oxLDL (U/ml)	3.82 ± 0.87	3.00 ± 0.92	−0.82 ± 0.74	−21.5%	<0.001
oxLDL/LDL (U/mg)	2.51 ± 0.54	2.20 ± 0.61	−0.31 ± 0.48	−12.4%	<0.001
oxLDL/ApoB (U/mg)	3.46 ± 0.72	3.22 ± 0.93	−0.21 ± 0.75	−6.0%	0.067
oxLDL/HDL (U/mg)	7.53 ± 2.72	5.71 ± 2.40	−1.82 ± 1.70	−24.2%	<0.001
hs-CRP (mg/L)	1.2 (0.5–2.0)	1.4 (0.7–2.2)	0.05 (−0.23–0.5)	4.2%	0.301

*The variables normally distributed are presented as mean ± SD, otherwise as median (IQR). EOT, end of treatment; FAU, fluorescence arbitrary units; hs-CRP, high sensitivity C-reactive protein.*

### Oxidative Stress

The difference in 4-HNE protein adducts in the serum was not statistically different after the 8-week treatment. OxLDL levels were lower both as absolute values (−21.5%, *p* < 0.001) (Figure 2B) and as oxLDL/LDL ratio (−12.4%, *p* < 0.001) (Table 4). There was a good correlation between the decrease in LDL and oxLDL (*r* = 0.438; *p* = 0.003). The change in the rate of apoptosis from baseline to end of treatment was related to the change in both oxLDL (*r* = 0.302, *p* = 0.041) and oxLDL/LDL ratio (*r* = 0.348, *p* = 0.018). On the other hand, it did not correlate with the change in LDL values (*r* = 0.003, *p* = 0.983).

**FIGURE 4:**
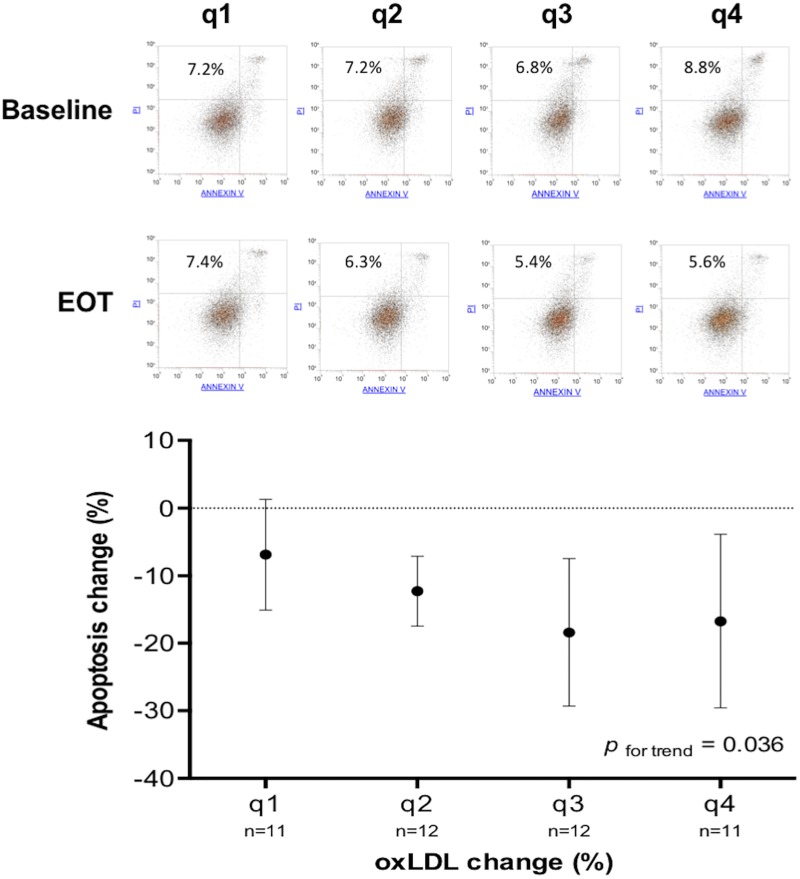
Change of apoptosis rate according to quartiles of oxLDL change. In the main graph, for each quartile of oxLDL change (q1 > −8.78%; q4 < −34.66%) is shown the mean and SD of apoptosis rate change. The upper part of the figure shows the flow cytometry and apoptosis rate (%) before and after treatment of four representative subjects belonging to different quartiles of oxLDL change.

The analysis of variance (ANOVA) of the apoptosis rate change among quartiles of the oxLDL change was statistically significant (*p* for trend = 0.036) (Figure 4).

### Safety

Nutraceutical compound was well tolerated and did not cause any change in creatinine, liver enzymes and creatine kinase values (Table 3).

## Discussion

Several NC based on red yeast rice with different dosages of monacolin K and other cofactors are available on the market. However, not of all them have been proven effective by proper scientific studies. This is relevant considering the variability in the quantity of monacolin K in 28 different red yeast rice supplements available in the United States of America (Cohen et al., 2017). When tested, the efficacy of these NC is determined by reduction of LDL cholesterol. Our study shows that the tested NC containing low monacolin K dose, polymethoxyflavones and antioxidants significantly reduced the LDL cholesterol after 8 weeks of treatment, thus confirming the previous studies (Ruscica et al., 2014; Mazza et al., 2015; Heinz et al., 2016; Pirro et al., 2016; Barrios et al., 2017). Our data also confirm the evidence that subjects with higher baseline values of LDL have the greatest benefit from NC treatment (Heinz et al., 2016).

The main strengths and novelties of our findings can be summarized as follows. First, we demonstrated that a NC with monacolin K, beside the reduction of LDL, improves endothelial function assessed *in vitro* as a reduction of the rate of apoptosis in HUVECs incubated with serum from treated subjects, a methodology that was previously validated and related to clinical outcomes (Agnoletti et al., 1999; Valgimigli et al., 2003; Campo et al., 2017; Pavasini et al., 2017). Enhanced endothelial apoptosis is supposed to be the initial step in the development of atherosclerosis. It is considered the underlying mechanism of the pleiotropic effects of both statins and ACE inhibitors, which have been shown to reduce endothelial apoptosis and increase endothelial NO production (Ceconi et al., 2007; Oesterle et al., 2017). In our study the NO levels in HUVECs were not significantly increased by the treatment with the tested NC. There are several explanations for this. The low-risk subjects enrolled in our study could have just a mild endothelial dysfunction with initial increase of the rate of apoptosis, but without significant impairment of endothelial NO production that could not be further improved by treatments, thus maintaining normal or near normal endothelial function. Furthermore, NO levels depend on cumulative effects of different factors, and endothelial cells apoptosis and NO are controlled by different pathways. Therefore it is not surprising that variations of serum cytokines or lipids may modulate apoptosis or NO pathway differently. Alternatively, the treatment duration of 8 weeks could be too short to result in an improvement in NO production.

Second, dyslipidaemia is known to be associated with oxidative stress and high oxLDL levels (Ercan et al., 2014; Harmon et al., 2016). Our study demonstrated that NC reduced oxLDL both in terms of absolute values and oxLDL/LDL ratio. In contrast, treatment did not reduce 4-HNE total protein adducts, a biomarker of oxidative damage to lipids. Low physiological levels of 4-HNE have a pro-survival role on endothelial cells, while high doses of 4-HNE result to be toxic (Chapple et al., 2013). Due to the lack of standardized data for 4-HNE adducts levels in serum, it is difficult to establish a 4-HNE physiological threshold. In line with the results of NO, this may be related to the low risk of the population studied with moderate dyslipidemia insufficient to cause abnormal accumulation of 4-HNE. This hypothesis is supported by the findings in which baseline 4-HNE levels do not correlate with LDL or other parameters related to dyslipidaemia and/or inflammation, suggesting that the majority of enrolled subjects could have 4-HNE physiological levels. The oxidation of LDL has been recognized as an early event in the progression of atherosclerosis, and the oxLDL levels are linearly associated with CV diseases (Trpkovic et al., 2015). It is known that apoptosis can be induced in endothelial cells by high levels of oxLDL via a mechanism mediated by the oxidized low-density lipoprotein receptor 1 (LOX1) (Valente et al., 2014) and carried out by classic caspases (Sata and Walsh, 1998). Indeed, we observed a correlation between the reduction of oxLDL and the reduction of apoptosis rate in HUVECs, suggesting that improvement in endothelial function may be mediated by a decrease in oxLDL. Hydroxytyrosol and vitamin E act as antioxidants protecting against LDL oxidation in humans (Najafpour Boushehri et al., 2012; Mateos et al., 2016), and nobiletin (polymethoxyflavone) and coenzyme Q10 can prevent endothelial cells apoptosis induced by oxLDL by modulating LOX-1 (Eguchi et al., 2006; Tsai et al., 2011). Overall, the effect on endothelial apoptosis is probably caused by the cumulative action on LDL lowering mediated by monacolin K, and the protection from LDL oxidation provided by the nutraceutical antioxidants.

Third, to our knowledge this is the first study on the use of a NC that selected the subjects using the most recent European Society of Cardiology (ESC) guidelines on dyslipidaemia and primary CV prevention (Catapano et al., 2016; Piepoli et al., 2016). Indeed, the selection of individuals was made considering both LDL value and the absolute CV risk of the subject, the latter calculated through SCORE. In literature, there are only two studies that included a CV risk score (Framingham) as an inclusion criterium (Gonnelli et al., 2015; Derosa et al., 2017). In all the others, subjects were enrolled only according to LDL values, with minimum baseline LDL values for inclusion ranging between 115 and 150 mg/dl with, sometimes, an upper limit, from 160 to 230 mg/dl. Choosing to start a preventive intervention based purely on a chemical or clinical value without considering the global risk of the subject could be non-optimal (Blood Pressure Lowering Treatment Trialists Collaboration, 2014; Stone et al., 2014; Catapano et al., 2016; Piepoli et al., 2016).

Fourth, unlike previous studies, we did not detect any change in hs-CRP. This can be explained with the lower dosage of monacolin K and coenzyme Q10 in our NC and, especially, with lower baseline hs-CRP values in our population. Indeed, when we performed a sub-analysis only in subjects with baseline hs-CRP greater than 2 mg/L [*N* = 10, 2.5 (2.1–3.5) mg/L)], there was a significant reduction after the treatment (−0.7 ± 0.6 mg/L, *p* = 0.008), in line with findings by Cicero et al. (2013, 2016a) (∼20%).

Fifth, subgroups analysis shows that subjects with metabolic syndrome had a lower reduction of LDL compared to subject without the syndrome, even though the difference is not significant. Many NC have been tested with success in subjects with metabolic syndrome, but we are not aware of studies of comparison between these groups (Ruscica et al., 2014; Verhoeven et al., 2015; Mazza et al., 2018). This finding is confirmed by a significant lower response to NC in subjects with abnormal WHR, an index of abdominal adiposity associated with metabolic abnormalities and increased CV risk (Emdin et al., 2017). However, NIRVANA was not powered to test this hypothesis, hence these findings have an exploratory value. A possible explanation could be that low adherence to healthy lifestyle recommendations had caused both the metabolic abnormalities (i.e., high WHR and/or metabolic syndrome) and a lower response to NC.

### Study Limitations

The present study has limitations. First, the absence of a control group; this decision was made because the efficacy of NC with 3 mg of monacolin K vs. placebo has already been investigated. Second, the study has a relatively small sample size, even though it was powered for the primary endpoint. Third, an 8-week study period, although in line with other studies, is not adequate to evaluate the long-term efficacy of NC. Fourth, in the view of previous experience of our team, potential effect of the NC on endothelial function has been tested with an *in vitro* experiment, rather than *in vivo* tests or biomarkers analysis. We are aware that the two latter should be preferred to the first one. However, the experiment we used was previously validated, and related to clinical outcomes. Finally, subgroups analysis and results on endothelial function have to be considered hypothesis generation. It follows that this is only a small “proof-of-concept,” hypothesis-generating study.

### Conclusion

The NIRVANA study showed that 8-week treatment with a NC containing low monacolin K dose, polymethoxyflavones, hydroxytyrosol, vitamin E, coenzyme Q10 and Ipomoea batatas extract was able to reduce by ∼15% LDL levels in subjects with dyslipidaemia and low to moderate CV risk. In addition, it was associated with a significant improvement in surrogate markers of endothelial function, mainly mediated by a reduction of oxLDL.

## Author Contributions

PC, GC, FVDS, FV, and PR contributed to conception and design of the study. PC, FV, VL, DB, GP, FF, GA, and LM organized the database. PC, GC, and VL performed the statistical analysis. PC, FVDS, and GC wrote the first draft of the manuscript. RF and PR wrote sections of the manuscript. All authors contributed to manuscript revision, read, and approved the submitted version.

## Conflict of Interest Statement

The authors declare that the research was conducted in the absence of any commercial or financial relationships that could be construed as a potential conflict of interest.

## Abbreviations

CV: cardiovascular
HNE: Hydroxynonena
HUVECs: human umbilical vein endothelial cells
LDL: low density lipoprotein
NC: nutraceutical compound
NO: nitric oxide
oxLDL: oxidized LDL
SCORE: systemic coronary risk estimation.
