# Supplementary material for: Effectiveness of a Novel Nutraceutical Compound Containing Red Yeast Rice, Polymethoxyflavones and Antioxidants in the Modulation of Cholesterol Levels in Subjects With Hypercholesterolemia and Low-Moderate Cardiovascular Risk: The NIRVANA Study
Source: Front Physiol. 2019 Mar 11;10:217. doi: 10.3389/fphys.2019.00217 (PMC6421317; doi:10.3389/fphys.2019.00217)
Supplement: Supplementary file 1 [file Data_Sheet_1.PDF]

Triglycerides change in different subgroups

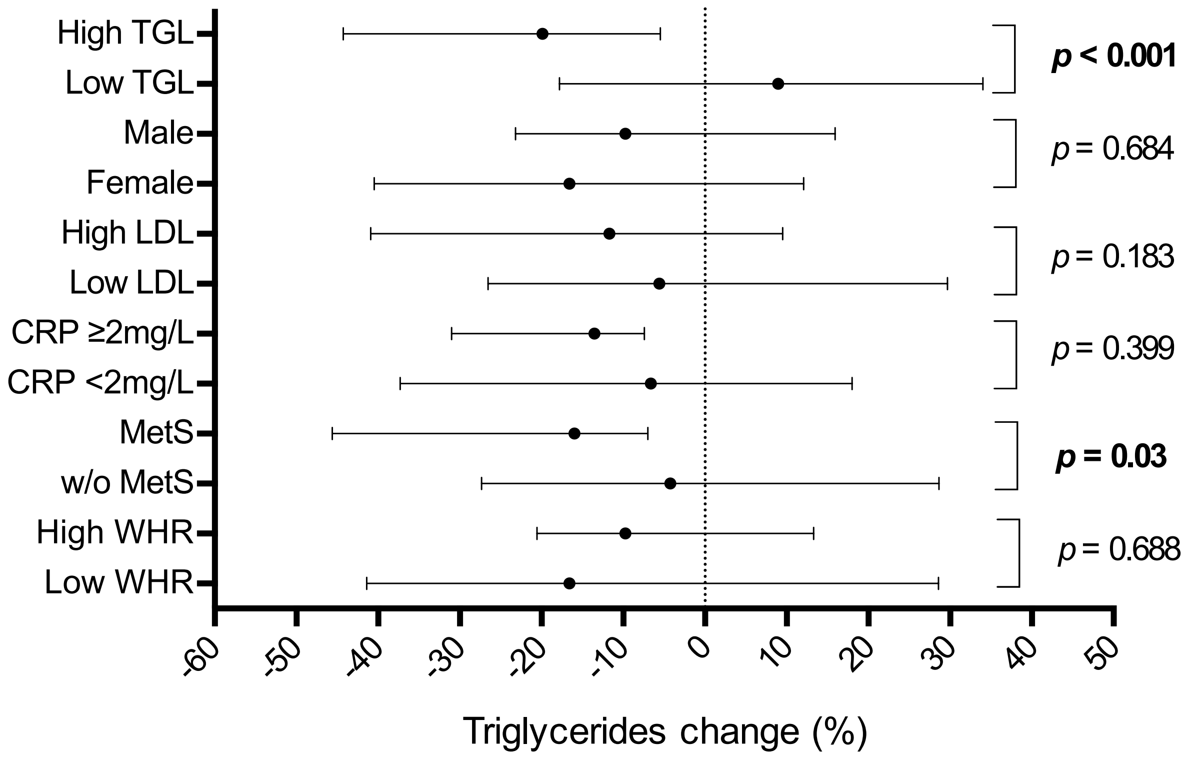

High/Low TGL is defined on median baseline values.
Triglycerides change is shown as median and interquartile range.
p-values refer to the difference in the subgroups shown on the left.
TGL: triglycerides. CRP: c-reactive protein. MetS: metabolic syndrome. WHR: waist-hip ratio.

HDL change in different subgroups

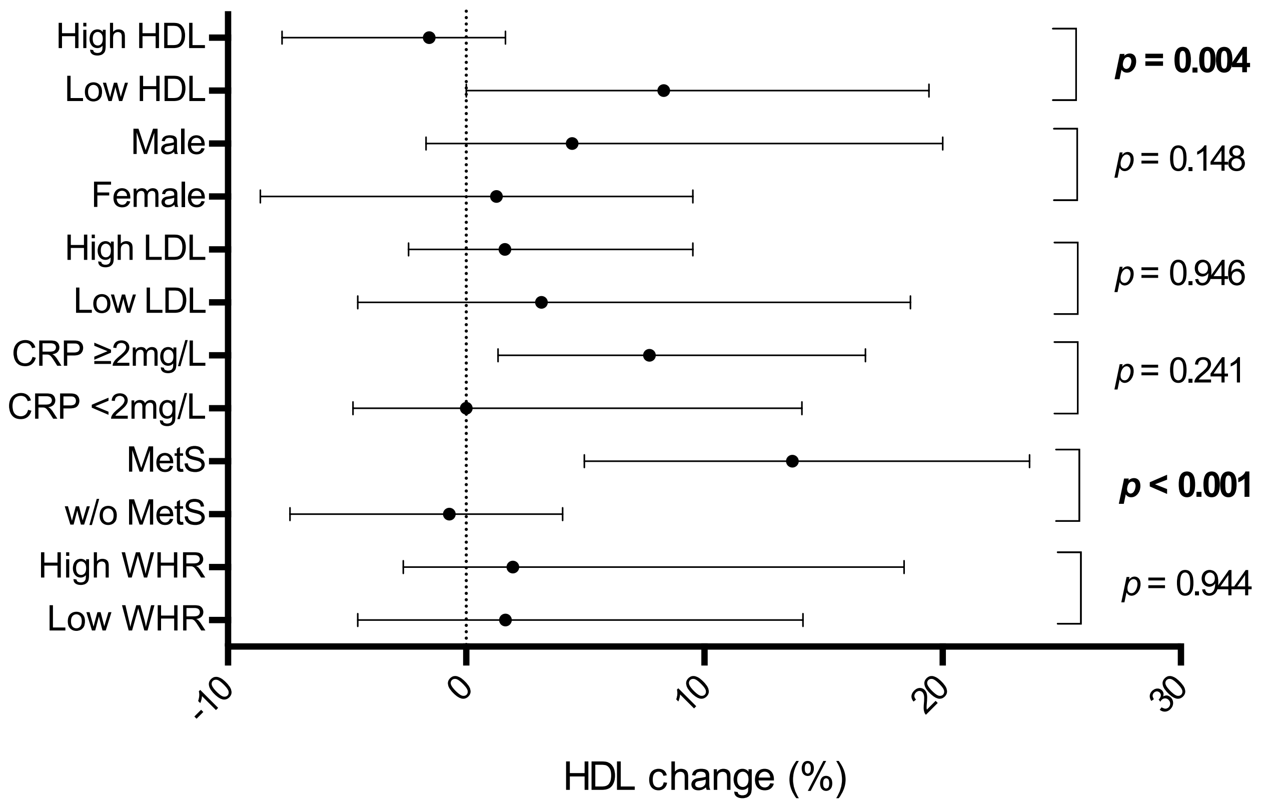

High/Low HDL is defined on median baseline values.
HDL change is shown as median and interquartile range.
p-values refer to the difference in the subgroups shown on the left.
CRP: c-reactive protein. MetS: metabolic syndrome. WHR: waist-hip ratio.

ApoB change in different subgroups

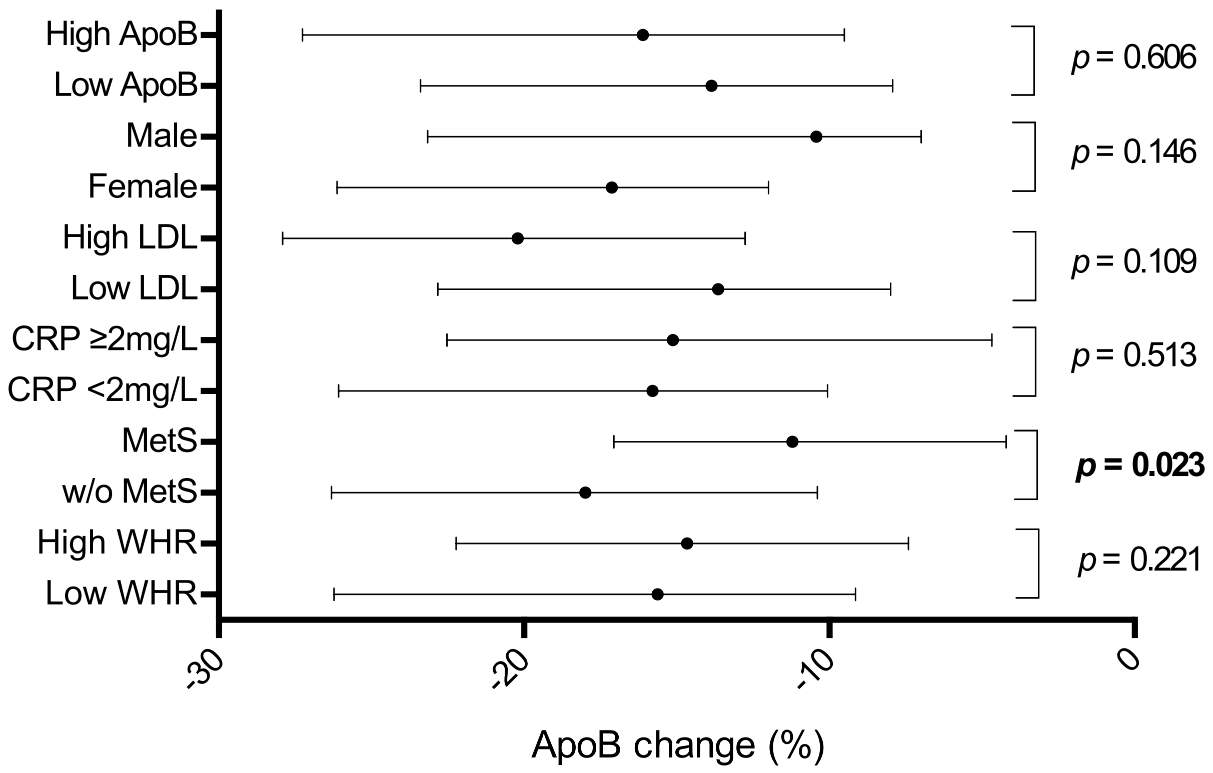

High/Low ApoB is defined on median baseline values.
ApoB change is shown as median and interquartile range.
p-values refer to the difference in the subgroups shown on the left.
CRP: c-reactive protein. MetS: metabolic syndrome. WHR: waist-hip ratio.

ApoA1 change in different subgroups

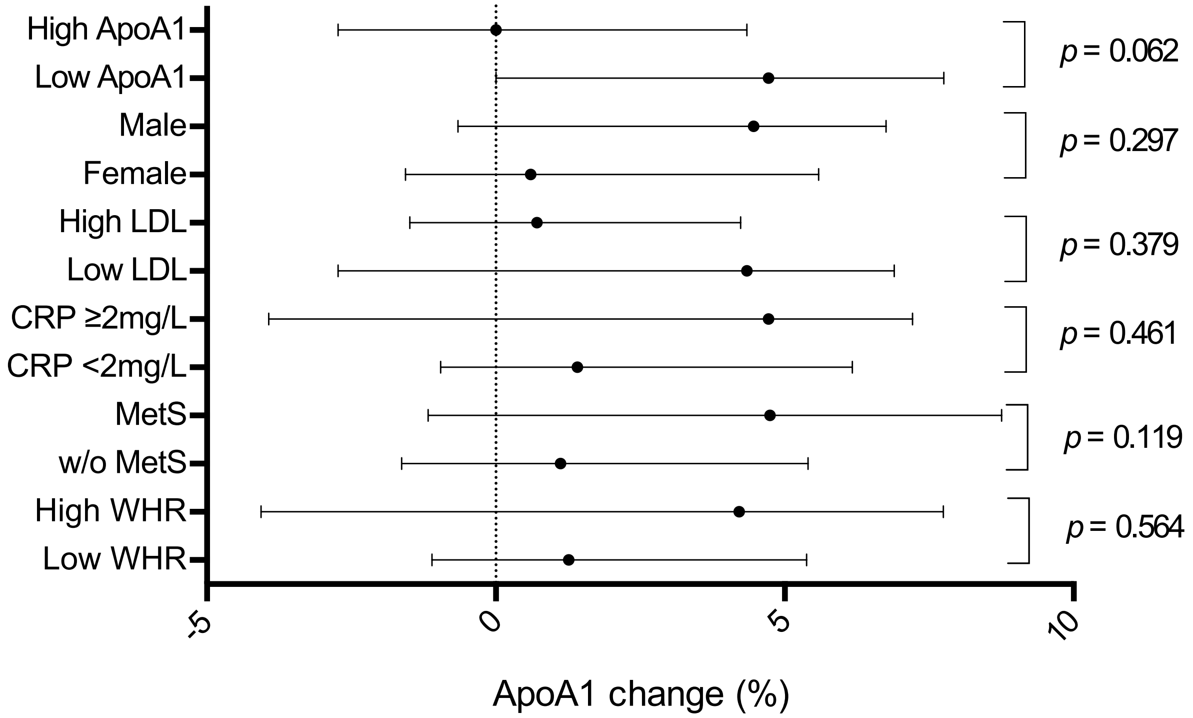

High/Low ApoA1 is defined on median baseline values.
ApoA1 change is shown as median and interquartile range.
p-values refer to the difference in the subgroups shown on the left.
CRP: c-reactive protein. MetS: metabolic syndrome. WHR: waist-hip ratio.
